# Neurosyphilis in China: A Systematic Review of Cases From 2009–2021

**DOI:** 10.3389/fmed.2022.894841

**Published:** 2022-05-13

**Authors:** Fang-Zhi Du, Hai-Ni Zhang, Jing-Jing Li, Zhi-Ju Zheng, Xu Zhang, Rui-Li Zhang, Qian-Qiu Wang

**Affiliations:** ^1^Department of Clinical Prevention and Control of STD, Institute of Dermatology, Chinese Academy of Medical Science and Peking Union Medical College, National Center for STD Control, China Centers for Disease Control and Prevention, Nanjing, China; ^2^Department of Dermatology, The Fifth People's Hospital of Suzhou, Suzhou, China; ^3^Department of Dermatology, The Second Affiliated Hospital of Nanjing Medical University, Nanjing, China

**Keywords:** neurosyphilis, clinical epidemiological characteristics, prevalence, systematic review, China

## Abstract

Considered the increased threaten of neurosyphilis in China, a review on cases reported in the literature to describe the clinical epidemiological characteristics of neurosyphilis cases, may be beneficial to the early detection and management strategies of neurosyphilis for clinicians. We searched the literature on Chinese neurosyphilis cases published from January 1, 2009 to December 31, 2021, described their clinical epidemiological characteristics and calculated the prevalence of neurosyphilis amongst other associated diseases, according to the individual study criteria. A total of 284 studies including 7,486 neurosyphilis cases were included. No meta-analysis was performed due to the heterogeneity of the data. Among 149 case reports and 93 retrospective case series studies, the main clinical manifestation of 3,507 neurosyphilis cases was cerebral parenchymal syphilis (57.3%), followed by asymptomatic neurosyphilis (16.7%), meningovascular syphilis (13.6%), meningitis syphilis (7.7%) and ocular syphilis (2.8%), etc. In addition, the initial diagnosis was incorrect in 53.2% patients, and the most frequent misdiagnoses were mental disorders (31.0%), stroke (15.9%), cognitive impairment (9.0%), etc. The positive or abnormal rates of cerebrospinal fluid non-treponemal and treponemal tests, white blood cell counts and protein concentrations were 74.2%, 96.2%, 61.5%, and 60.9%, respectively. Aqueous penicillin was the first choice for treatment in 88.3% cases, and 81.7% and 50.0% patients had response in the improvement of symptoms and serological effective in CSF, respectively. Among 26 studies on neurosyphilis patients amongst other associated diseases, the prevalence of neurosyphilis amongst central nervous system infectious diseases, syphilis-associated neurological symptoms, serofast status, coinfected with human immunodeficiency virus were 10.6%–30.1%, 23.2%–35.5%, 9.8%–56.1%, and 8.9%, respectively. In summary, the lack of early detection of neurosyphilis cases remains a clinical challenge. The high rate of misdiagnosis and high prevalence of neurosyphilis amongst associated diseases strongly remind clinicians to focus on the early detection among suspected cases. Besides, the standard treatment regimen and long-term follow-up, which complied with guideline should be provided. Further prospective studies are urgent to better delineate the clinical epidemiological characteristics of neurosyphilis in China.

## Introduction

Neurosyphilis, historically caused by *Treponema pallidum* (*T. pallidum*) infection, was reported increasing with the expansion of syphilis screening in China. *T. pallidum* invades the central nervous system (CNS) and may cause severe and irreversible neurologic sequelae in patients if left untreated (1). According to the latest report, the number of newly reported cases of syphilis was 438,199 (32.2 per 100,000) in 2016 and increased by an annual average of 8.6% from 2007 to 2016 in China; moreover, the number of reported cases of tertiary syphilis increased by 8.0% annually from 2007 to 2016 (2). Previous studies showed that the epidemiology of neurosyphilis largely paralleled that of syphilis (3, 4), and most tertiary syphilis cases were diagnosed as neurosyphilis (5), which indicated an increasing incidence of neurosyphilis in China.

Neurosyphilis has puzzled dermatologists, neurologists and psychiatrists in clinical settings for over two centuries because of its atypical symptoms and lack of a golden criteria of diagnosis. Early injury to CNS in neurosyphilis patients affects the mesenchyma, such as the meninges and blood vessels, manifesting within months to the several years after primary infection as meningismus, blindness, stroke, etc., while late injury affects the brain and spinal cord parenchyma within years to decades and presents as general paresis and tabes dorsalis (6, 7). Therefore, the rates of misdiagnosis and missed diagnosis in clinical settings are relatively high because of the diverse and atypical symptoms (8, 9). It is necessary for clinicians to be aware of the most common misdiagnosed diseases and specific clinical features of neurosyphilis when making a differential diagnosis.

Clinical recognition of neurosyphilis depends on the comprehensive assessment of clinical characteristics and cerebrospinal fluid (CSF) findings. However, no single specific and sensitive test for neurosyphilis exists. CSF pleocytosis and elevated protein concentrations are frequently observed in patients with neurosyphilis. Reactive CSF serologic tests are required for the diagnosis of neurosyphilis, and the Venereal Disease Research Laboratory (VDRL) test for CSF is thought to be the gold standard for specificity in the absence of blood contamination, but its sensitivity is still debated (10). The rapid plasma reagin (RPR) test, toluidine red unheated serum test (TRUST), fluorescent treponemal antibody adsorption (FTA-ABS) test and treponema pallidum particle agglutination (TPPA) test for CSF have all been assessed to have variable sensitivity and specificity in diagnosing neurosyphilis (11–13). Besides, no VDRL kits in China gets the approved of State Food and Drug Administration (SFDA) to date. Together the above all, the RPR and TRUST were recommended as the alternative tests by the ‘China National Guidelines for the Diagnosis and Treatment of Syphilis, Gonorrhea and Chlamydia Trachomatis Infection (2020)' (14). The diagnostic criteria for neurosyphilis differed between studies due to the lack of a gold standard test. Consequently, the methodological quality of neurosyphilis diagnosis among studies is unknown and need to be evaluated.

Although many cases of neurosyphilis in China were reported, their clinical features and management information have not reviewed comprehensively to date. In this paper, all cases of neurosyphilis reported in China in the past 13 years were reviewed, and their clinical epidemiological characteristics were presented, which will be helpful to clinicians to early detection and management of neurosyphilis.

## Materials and Methods

The research and reporting methods of this review were consistent with the preferred reporting items for systematic reviews (PRISMA) (Supplementary Table S1).

### Search Strategy

We searched the PubMed, EMBASE, and some Chinese Journal databases including China national knowledge infrastructure (CNKI) and WanFang databases for studies on neurosyphilis in China and limited the search to studies published between 1 January 2009 to 31 December 2021.We searched for ((“CSF” OR “lumbar puncture” OR “meningitis” OR “meningovascular” OR “stroke”) AND “syphilis”) OR (“neurosyphilis” OR “tabes dorsalis” OR “general paresis”) AND (“China”) in the PubMed and EMBASE databases and searched for “neurosyphilis” in the CNKI and WanFang databases (15). No language restrictions were set.

### Inclusion/Exclusion Criteria

We selected unduplicated references and excluded reviews if the studies did not address neurosyphilis, did not describe the clinical features of neurosyphilis patients, reported patients already described in a different paper, did not include Chinese patients, did not report new primary material or could not be downloaded. We did not limit inclusion based on the diagnostic criteria used for neurosyphilis.

### Selection Process

**Figure 1:**
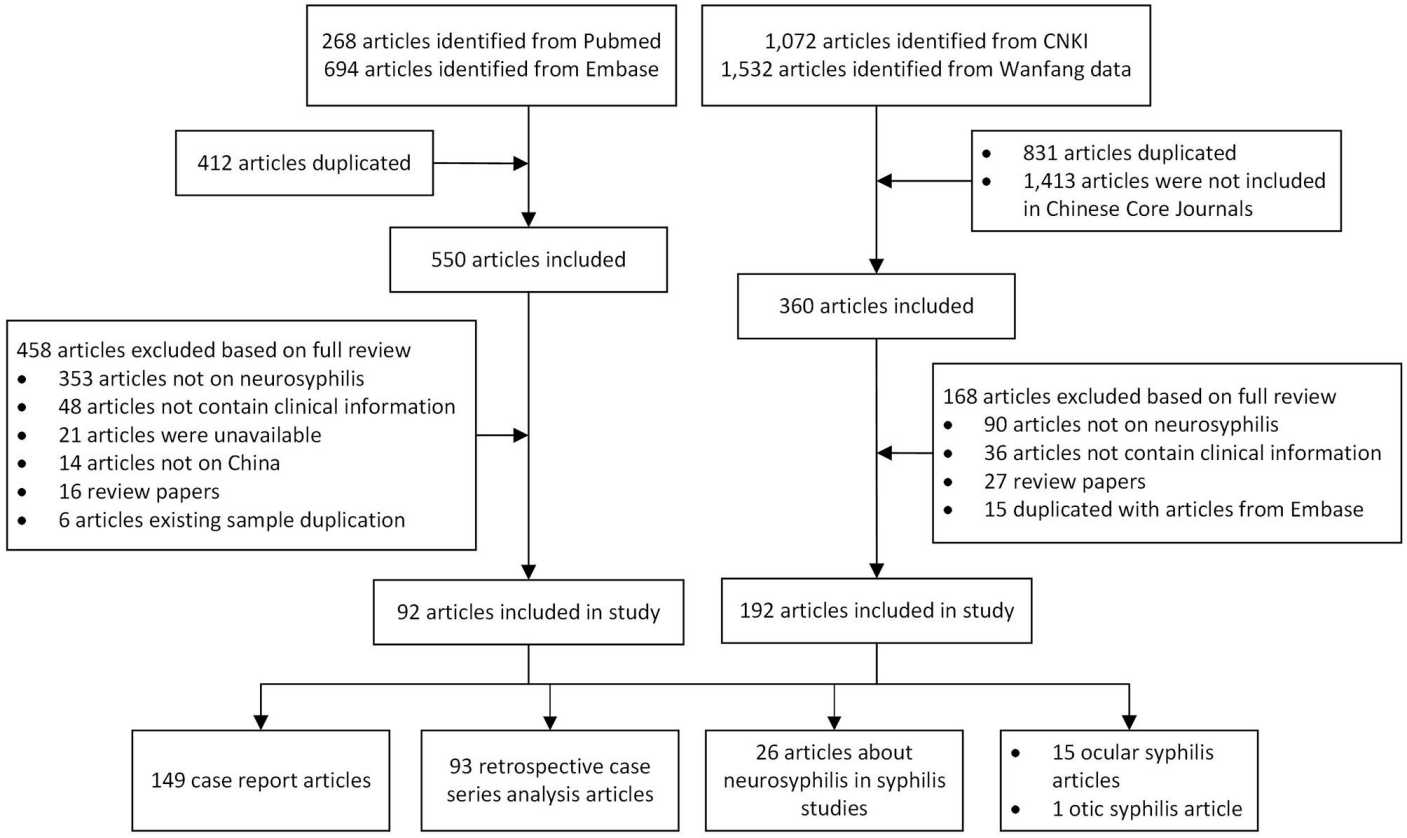
Study inclusion flowchart.

The selection was conducted by two independent reviewers working in parallel, first excluded duplication literature, then screening for title and abstract followed by the full text according to the inclusion/exclusion criteria. After the screening of 50 references, a validation of the screening process was conducted by comparing the screening results and discussing them within the team. Throughout the screening process, discrepancies were discussed and a third reviewer was consulted if consensus was not reached. The reasons for exclusion were documented only for full text publications (Figure 1).

### Data Extraction

We extracted variables including available demographic information of neurosyphilis patients, duration of the study, inclusion criteria, diagnostic criteria for neurosyphilis, clinical syndromes and misdiagnosis, neuroimaging findings, and the number of human immunodeficiency virus (HIV) -infected patients in each study. We also extracted information on the treatment and follow-up of patients in these studies.

### Statistical Analysis

For case reports and retrospective case series, we analyzed the sex ratio, age distribution, clinical spectrum, misdiagnosis rate, treatment, and the proportion of anomalies of CSF tests and neuroimaging findings. For studies on neurosyphilis in patients with syphilis or HIV, we calculated the proportion of study participants who were diagnosed with neurosyphilis according to the individual study criteria. No meta-analysis was performed due to the heterogeneity of the data.

The chi-squared test was used to compare categorical variables. The Wilcoxon signed-rank test was used to compare related continuous variables with skewed distributions. A *P*-value of < 0.05 was considered statistically significant. All statistical analyses were conducted in SPSS 21.0 (IBM Corp, Armonk, NY).

## Results

A total of 962 articles were identified in the PubMed and EMBASE databases, and 412 duplicated articles were excluded. Among the remaining 550 articles, 458 articles were excluded based on full-text reviews. A total of 2,604 articles were identified from the CNKI and Wanfang databases, and 831 duplicated articles and 1,413 articles that were not included in Chinese Core Journals were excluded. Among the remaining 360 articles, 192 articles were excluded based on full-text reviews. Finally, 284 articles that met our inclusion criteria were selected (Figure 1).

### Clinical Characteristics of Cases

The 149 case reports included 180 patients, consisting of 128 males and 52 females. The average age was 47.7 ± 12.9 (range from 12 to 79 years old), and the age of most patients ranged from 41 to 60 (56.7%, 102/180). The 93 retrospective case series included 3,327 patients (2,520 males, 783 females and 24 unknown), and neurosyphilis was more likely to occur in males than in females (|Z| = 8.24, P < 0.001) (Supplementary Table S2).

As shown in Table 1, among the 3,200 patients with clearly defined clinical subtypes, 533 (16.7%) has asymptomatic neurosyphilis, 726 (22.7%) had mesenchymal syphilis (245 (7.7%) had meningitis syphilis, 435 (13.6%) had meningovascular syphilis and 46 had undefined syphilis), 1,832 (57.3%) had parenchymal neurosyphilis (1,537 (48.0%) had general paresis, 115 (3.6%) had tabes dorsalis, 54 (1.7%) had syphilitic gumma and 126 had undefined syphilis), 90 (2.8%) had ocular syphilis, 42 (0.6%) had mixed-type syphilis, and 210 had undefined syphilis. The initial diagnosis was correct in 774 (46.8%) patients and incorrect in 847 (53.2%) patients among 173 studies, the correct diagnostic rate ranged from 17.0 to 100% (95%CI, 59.1–76.2%). Among the 847 misdiagnosed patients, 578 patients showed the misdiagnosed diseases, including mental disorders (179 patients, 31.0%), stroke (92 patients, 15.9%), cognitive impairment (52 patients, 9.0%), encephalitis (46 patients, 8.0%), ophthalmic diseases (40 patients, 6.9%), Alzheimer's disease (AD) (22 patients, 3.8%), brain tumors (20 patients, 3.5%), epilepsy (13 patients, 2.3%), myeleterosis (12 patients, 2.1%), peripheral neuropathy (11 patients, 1.9%), demyelinating disease or multiple sclerosis (10 patients, 1.7%), Parkinson's disease (PD) (nine patients, 1.6%), hydrocephalus (six patients, 1.0%), ataxiaor menopausal symptom (four patients, 0.7%, respectively), encephalatrophy (three patients, 0.5%), and other diseases (55 patients, 9.5%).

**Table 1:** Clinical characteristics of neurosyphilis cases from 149 case reports and 93 retrospective case series.

**Clinical characteristics**				**Number** **of cases**	**Proportion** (%) ^*^
		Asymptomatic		533	16.66%
		Mesenchymal syphilis	Meningitis	245	7.66%
			Meningovascular	435	13.59%
			Not mentioned	46	1.44%
Clinical subtypes	196 studies mentioned	Parenchymal syphilis	General paresis	1,537	48.03%
			Tabes dorsalis	115	3.59%
			Syphilitic gumma	54	1.69%
			Not mentioned	126	3.94%
		Ocular syphilis		90	2.81%
		Mixed		19	0.59%
		Not mentioned		97	-
	46 studies (28 case reports) not mentioned			210	-
Diagnosis	correct diagnosis			744	46.76%
	173 studies misdiagnosed diseases mentioned	Mental disorders		179	30.97%
		Stroke		92	15.92%
		Encephalitis		46	7.96%
		Cognitive impairment		52	9.00%
		ophthalmic diseases		40	6.92%
		Brain tumor		20	3.46%
		Alzheimer's disease		22	3.81%
		Epilepsy		13	2.25%
		Myeleterosis		12	2.08%
		Peripheral neuropathy		11	1.90%
		Parkinson's disease		9	1.56%
		Demyelinating disease and multiple sclerosis		10	1.73%
		Ataxia		4	0.69%
		Hydrocephalus		6	1.04%
		menopausal symptom		4	0.69%
		Encephalatrophy		3	0.52%
		Others		55	9.52%
		Not mentioned		269	-
	69 studies (21 case reports) not mentioned			1,916	-

* *The denominator is the sum of the cases who mentioned the clinical characteristics*.

### Laboratory Characteristics and Auxiliary Examination of Cases

Among 180 patients from 149 case reports studies, nontreponemal tests (VDRL/RPR/TRUSTs) were performed in 83.3% (150/180) of the patients, and 82.0% (123/150) of them patients had positive results. Treponemal tests (TPPA/TPHA) were performed in 78.3% (141/180), and 95.0% (134/141) of them had positive results. As shown in Table 2, patients were more likely to have positive results on the treponemal tests than non-treponemal tests (χ^2^ = 11.97, *P* = 0.001). However, there were no differences in the abnormal CSF white blood cell counts (WBC) rate (74.6%, 82/110) and abnormal CSF protein (PRO) rate (78.9%, 86/109) between these two groups (χ^2^ = 0.58, *P* = 0.52). We also found that 24 patients were diagnosed with neurosyphilis without reactive CSF serologic tests. (Supplementary Table S1).

**Table 2:** Laboratory findings of neurosyphilis cases from 149 case reports and 93 retrospective case series.

**Laboratory findings**			**Number of cases**	**Positive or abnormal proportion** (%) *	***χ^2^***	**|Z|^†^**	***P***
CSF serological test	Case reports	VDRL/RPR/TRUST-positive	123	82.00%	11.97		0.001
		FTA-ABS/TPPA/TPHA-positive	134	95.04%			
		WBC count abnormal	82	74.55%	0.58		0.523
		PRO level abnormal	86	78.90%			
	Retrospective case series	VDRL/RPR/TRUST-positive	1,747	73.71%		5.78	<0.001
		FTA-ABS/TPPA/TPHA-positive	2,339	96.22%			
		WBC abnormal	1,654	60.99%		1.1	0.267
		PRO level abnormal	1,665	60.22%			
Neuroimaging examination (MRI/CT)	215 studies mentioned	Abnormal	1,750	81.89%			
		Normal	387	18.11%			
		Not mentioned	475				
	27 studies (12 case reports) not mentioned	897				
EEG	50 studies mentioned	Abnormal	458	77.50%			
		Normal	133	22.50%			
		Not mentioned	494				
	192 studies (129 case reports) not mentioned	2,422				
HIV	135 studies mentioned	Positive	228	8.34%			
		Negative	2,505	91.66%			
	107 studies (73 case reports) not mentioned	774				

CSF, cerebrospinal fluid; VDRL, venereal disease research laboratory; RPR, rapid plasma reagin; TRUST, toluidine red unheated serum test; FTA-ABS, fluorescent treponemal antibody adsorption; TPPA, treponema pallidum particle agglutination; TPHA, treponema pallidum hemagglutination assay; WBC, white blood cell; PRO, protein; MRI, magnetic resonance imaging; CT, computed tomography; EEG, electroencephalography; HIV, human immunodeficiency virus. *The denominator is the sum of the cases who mentioned the laboratory findings.

† *The Wilcoxon signed-rank test was used to compare the difference in positive rate between CSF non-treponemal tests and treponemal tests, as well as the abnormal rate between CSF WBC and PRO level*.

Among the 93 retrospective case series studies, non-treponemal tests (VDRL/RPR/TRUST) were performed in 88.8% (2,491/2,805) of the patients, and 73.7% (1,747/2,370) of them had positive results, the positive rates ranged from 0 to 100% (95%CI, 69.5–81.2%). Treponemal tests (FTA-ABS/TPPA/TPHA) were performed in 91.0% (2,552/2,805) of the patients, and 96.2% (2,339/2,431) of them had positive results, the positive rates ranged from 66.7 to 100% (95%CI, 90.8–97.3%). Patients were more likely to have a positive result on a treponemal test than a nontreponemal test (|Z = 5.78, *P* < 0.001). CSF-WBC were abnormal among 61.0% (1,654/2,712) patients, the abnormal rates ranged from 22.2% to 100% (95%CI, 59.0–69.7%). CSF-PRO test was abnormal among 60.2% (1,665/2,765) patients, the abnormal rates ranged from 11.1 to 100% (95%CI, 65.1–75.8%). There were no differences in the rates of abnormal CSF-WBC and CSF-PRO results among studies (|Z| = 1.1, *P* = 0.27). The diagnostic criteria for neurosyphilis were not provided in two studies including 51 patients, and the results of CSF serologic tests were not mentioned in 12 studies including 474 patients. (Supplementary Table S3).

The numbers of patients with HIV infection were described in 135 studies, including 2,733 patients, and the coinfection rate was 8.34% (228/2,733). In addition, imaging examination results were presented in 215 studies; 81.8% (2,137/2,612) of the patients underwent neuroimaging examinations (skull/spine/orbital magnetic resonance imaging (MRI) or computed tomography (CT)), and 81.9% (1,750/2,137) of them had abnormal results (Table 2), the abnormal rates ranged from 25.0 to 100% (95%CI, 80.5–88.2%). Forty-two studies presented electroencephalography (EEG) findings; 54.47% (591/1,085) of the patients underwent EEG, and 77.50% (458/591) of them had abnormal results, the abnormal rates ranged from 11.5% to 100% (95%CI, 70.6–87.8%) (Supplementary Table S3).

### Treatment and Prognosis

Of the 242 studies, treatment of neurosyphilis was described in 201 studies including 1,738 patients. As shown in Table 3, the treatment drugs were described for 1,634 patients. Aqueous penicillin was the first choice for treatment (88.3%, 1,442/1,634), and ceftriaxone (7.4%, 121/1,634) and doxycycline (0.5%, 8/1,634) were the alternative choices for those who were allergic to penicillin. The remaining 63 (3.9%) patients were not treated with the drugs recommended by the National Guidelines, such as benzathine penicillin (3.2%, 53/1,634), minocycline, azithromycin, traditional Chinese medicine (TCM), etc. Thirty eight patients were referred to other hospitals and 10 patients refused treatment.

**Table 3:** The treatment and follow-up of neurosyphilis cases from 149 case reports and 93 retrospective case series.

**Treatment and prognosis**				**Number of cases**	Proportion (%) *
Treatment drugs		Aqueous penicillin		1,442	88.25%
		Ceftriaxone		121	7.41%
		Doxycycline		8	0.49%
	201 studies mentioned	Benzathine penicillin		53	3.24%
		Other drugs		10	0.61%
		Refused treatment		10	-
		Referred		38	-
		Not mentioned		56	-
	41 studies (11 case reports) not mentioned			1,769	-
		Symptoms	improved	650	68.06%
			recovery	130	13.61%
			no-response (persisted, deteriorated, or recurrence)	175	18.32%
Follow-up and Prognosis	181 studies mentioned	Serum non-treponemal test dropped at least 2 titers or turn to negative		195/288	67.71%
		CSF non-treponemal test dropped at least 2 titers or turn to negative		89/178	50.00%
		CSF WBC decreased		145/188	77.13%
		CSF PRO decreased		153/202	75.74%
		Neuroimaging improved		35/36	97.22%
		Death		9	-
	20 studies (9 case reports) not mentioned		679	-

CSF, cerebrospinal fluid; WBC, white blood cell; PRO, protein. *The denominator is the sum of the cases who mentioned the treatment and prognosis.

The follow-up and prognosis of patients were described in 181 studies. The longest follow-up time was 6 years, while the efficacy of treatment among 19.3% (29/150) patients were evaluated before discharge and only 17.3% (26/150) patients follow-up at least 1 year. Most patients (52%, 78/150) follow-up for 3 to 6 months. As shown in Table 3, during the follow-up period, 81.7% (780/955) patients had an improvement or recovery of clinical symptoms (range from 0 to 100% in 47 cases series studies, 95%CI: 75.0–88.3%, Supplementary Table S2). Unfortunately, the persistence, deteriorate or recurrence of symptoms were occurred among 18.3% (175/955) patients. The results of nontreponemal test in serum or CSF turned negative or dropped by at least two titers in 67.7% (195/288) (range from 0 to 100% in 14 cases series studies, 95%CI: 33.0%−73.3%, Supplementary Table S2) and 50% (89/178) (range from 0 to 100% in 10 cases series studies, 95%CI: 23.2–73.7%, Supplementary Table S2) of patients, respectively. The CSF WBC or PRO decreased or return to normal range in 77.1% (145/188) (range from 42.9 to 100% in 12 cases series studies, 95%CI: 64.5–94.8%, Supplementary Table S2) and 75.7% (153/202) (range from 45.5 to 100% in 10 cases series studies, 95%CI: 64.8–93.4%, Supplementary Table S2) of patients, respectively. In addition, the rate of improvement or recovery on neuroimaging findings was 97.2% (35/36) mentioned in 34 case report studies. Nine patients died during follow-up (Supplementary Tables S2, S3).

Among 121 cases report studies, 131 and 13 patients were mentioned treated by using aqueous penicillin or ceftriaxone, and the response to clinical symptoms after treatment were mentioned in 107 and 12 cases, respectively. We found that there is no difference in the rate of improvement or recovery of clinical symptoms between patients treated by using aqueous penicillin or ceftriaxone (87.9% (94/107) vs. 83.3% (10/12), *P* = 1.0). Data about the syphilitic serological response rate in serum and CSF was too few for comparison.

### Neurosyphilis Amongst Patients With Other Associated Diseases

Seven studies reported cases of neurosyphilis amongst CNS diseases (Table 4, Supplementary Table S3). The diagnostic criteria of neurosyphilis were not stated in five studies, and the results of CSF serological tests were presented in only one study. Twenty one studies reported cases of syphilis with neurosyphilis (Table 5). The performance of LP was mentioned in all studies, however, the CSF serologic tests were not performed among 238 patients in three studies.

**Table 4:** Studies reporting cases of neurosyphilis with CNS diseases.

**ID**	**Study**	**Province**	**Year**	**Neurosyphilis**	**Prevalence of neurosyphilis**	**Study design**	**Study Duration**	**Inclusion criteria**	**CSF criteria for diagnosis of neurosyphilis**	**% HIV positive**
1	Dai LL ^18^	Beijing	2014	2	3.33%	retrospective	2009-2011	HIV/AIDS patients (≥13 years old) with a complaint of new or recurrent neurological or psychiatric symptoms/signs	Not Stated	100.00%
2	Guan LQ ^19^	Shanghai	2016	36	10.62%	retrospective	2010-2015	HIV with CNS lesions	Positive result on CSF-VDRL, or FTA-ABS tests with abnormal CSF-WBC	100.00%
3	FF Yu^20^	Tianjin	2021	11	22.92%	retrospective	2017-2020	HIV with CNS diseases	Not Stated	100.00%
4	Lv LX ^21^	Tianjin	2015	1	Syphilis: 0.22%	retrospective	2011-2014	Inpatient with nervous system disease	Not Stated	Not Stated
					CNS diseases: 0.0028%					
5	Qin LH ^22^	Guangxi	2014	1	1.43%	retrospective	2003-2012	HIV with neuropathy	Not Stated	100.00%

*CNS, central nervous system; CSF, cerebrospinal fluid; HIV, human immunodeficiency virus; AIDS, acquired Immune Deficiency Syndrome; VDRL, venereal disease research laboratory; FTA-ABS, fluorescent treponemal antibody adsorption; WBC, white blood cell*.

**Table 5:** Studies on neurosyphilis among patients with syphilis.

**ID**	**Study**	**Province**	**Year**	**Neurosyphilis**	**Prevalence of Neurosyphilis**	**Study design**	**Study Duration**	**Inclusion criteria**	**CSF Criteria for diagnosis of neurosyphilis**	**% HIV +**
1	Li K ^23^	Shanghai	2013	100	23.70%	retrospective case-control	2009–2012	Syphilis LP performed	CSF-VDRL (87 cases) and TPPA (13 cases) reactivity	0.00%
2	Xiao Y ^24^	Fujian	2017	123	33.24%	retrospective	2008–2014	HIV-negative with neurological symptoms	CSF-RPR (123 cases) reactivity	0.00%
3	Zhang L ^25^	Guang dong	2010	156	35.45%	clinical trial	2007–2010	Syphilis diagnosed in a dermatology clinic	VDRL/FTA-ABS (156 cases) reactivity	Not Stated
4	Zhu L ^26^	Shanghai	2014	210	13.63%	retrospective	2009–2012	Syphilis in patients ≥18 years old	Positive CSF-TPPA in the absence of contamination with blood or CSF-VDRL reactivity (210 cases)	0.00%
5	Shi M ^27^	Shanghai	2016	191	22.90%	retrospective	2009–2013	HIV-negative syphilis	(1) CSF-VDRL reactivity or nonreactivity (117 cases) and (2) an elevated CSF-protein level (>50 mg/dL) or WBC (>10 cells/L) in the absence of other known causes of the abnormalities	0.00%
6	Ma CD ^28^	Jiangsu	2013	7	26.92%	retrospective	2007–2011	Inpatients with syphilis	CSF-RPR (7 cases) and TPHA (13 cases) reactivity	Not Stated
7	Lin DH ^29^	Fujian	2017	222	61.16%	retrospective	2005–2013	Syphilis LP performed	CSF-RPR (92 cases) or TPPA reactivity and WBC count or PRO level abnormality	0.00%
8	Li SL ^30^	Fujian	2012	115	56.10%	retrospective	2006–2009	Serofast syphilis	CSF-TRUST (58 cases) and TPPA (93 cases) reactivity	0.00%
9	Cai SN ^31^	Beijing	2017	139	34.58%	retrospective	2008–2016	Serofast syphilis	Presence of one or more CSF abnormalities (pleocytosis, elevated protein concentration, or CSF-RPR reactivity (40 cases))	0.00%
10	Zheng TH ^32^	Guang dong	2016	6	9.84%	cost-benefit analysis	2013	Serofast syphilis	CSF-RPR (6 cases) reactivity and an abnormal CSF-WBC	Not Stated
11	He WQ ^33^	Guang dong	2015	12	26.09%	retrospective case-control	Not Stated	Serofast syphilis	CSF-FTA-ABS (5 cases) or CSF-TPHA (11 cases) reactivity	0.00%
12	Ye YJ ^34^	Zhejiang	2018	127	27.79%	clinical trial	2012–2015	Serofast syphilis	CSF-VDRL (67 cases), CSF-RPR (73 cases), CSF-TPPA (252 cases), or CSF-FTA-ABS (244 cases) reactivity	0.44%
13	Chen XS ^35^	Guang dong	2011	6	13.64%	retrospective	2002–2009	Syphilis patients whose RPR titer increased by 2 times or more without reinfection after standardized treatment	CSF-RPR and TPPA (1 case) and CSF-RPR and TPPA and VDRL (5 cases) reactivity	Not Stated
14	Wang YJ ^36^	Taiwan	2012	14	8.92%	retrospective	2000–2009	HIV and syphilis coinfection	CSF-WBC >20 cells/μL (7 cases) or elevated VDRL titers in CSF samples (7 cases)	100.00%
15	XX Sun^37^	Henan	2020	51	50.00%	retrospective	2014-2017	CSF abnormal and HIV-coinfection	CSF TPPA (51 cases) and RPR (15 cases) reactivity and abnormal CSF-WBC and PRO	100.00%
16	Zhu L ^38^	Shanghai	2019	7	26.92%	retrospective	2008–2018	Malignant syphilis	CSF-VDRL (7 cases) reactivity	14.29%
17	Tang WM ^5^	Guang dong	2017	1615	0.54%	retrospective	2009–2014	Syphilis LP performed	CSF-VDRL reactivity or a CSF WBC > 20 cells/μL	Not Stated
18	Wang H ^39^	Sichuan	2011	24	1.25%	retrospective	2006–2010	Inpatient status	CSF-TRUST (24 cases) reactivity and an abnormal CSF-WBC and CSF-PRO level	Not Stated
19	J Yan^40^	Beijing	2021	416	78.79%	retrospective	2013-−2019	patients >18 years old; laboratory-confirmed syphilis in Department of Neurology or HIV infection	reactive CSF TPPA or TRUST, CSF WBC ≥5 cells/μL for HIV-negative patients and >20 cells/μL for HIV-positive, or elevated protein (>500mg/L); if CSF TPPA or TRUST was not reactive, no evidence of other diseases of the CNS could cause CSF pleocytosis or elevated protein.	73.08%
20	J Cao^41^	Xinjiang	2021	10	13.70%	retrospective	2016–2019	Serofast syphilis, or coinfection with HIV, or with neurological symptoms, or serum titer 4 times fluctuation	CSF TPPA (10 cases) reactivity, or CSF TRUST (5 cases) reactivity and CSF WBC or PRO abmormal	10.00%
21	YH Hua^42^	Jiangsu	2021	121	23.63%	retrospective	2016–2019	Serofast syphilis, or with neurological symptoms, or serum titer fluctuation	CSF TPPA (121 cases) reactivity, or CSF TRUST (77 cases) reactivity and CSF WBC (59 cases) or PRO (68 cases) abmormal	Not Stated

*CSF, cerebrospinal fluid; LP, lumbar puncture; VDRL, venereal disease research laboratory; TPPA, treponema pallidum particle agglutination; HIV, human immunodeficiency virus; RPR, rapid plasma reagin; FTA-ABS, fluorescent treponemal antibody adsorption; WBC, white blood cell; PRO, protein; TRUST, toluidine red unheated serum test; CNS, central nervous system*.

#### CNS Diseases

A case-control study in patients with CNS infection showed that 30.1% (84/279) of patients were diagnosed as neurosyphilis with evidence of positive reaction on CSF serological tests (16). Another study in patients with cognitive impairment showed that neurosyphilis was prevalent in 17.1% (6/35) of the patients with CNS infectious diseases; however, the diagnostic criteria of neurosyphilis were not provided (17). Other three studies reported neurosyphilis amongst HIV-infected patients with CNS disorders. There were 60, 339, and 48 patients recruited, and 2, 36 (10.6%) and 11(22.9%) of them were diagnosed with NS, respectively (18–20). However, only one study (19) provided evidence in positive results on the CSF-VDRL test or FTA-ABS test and abnormal CSF-WBC for the diagnosis of neurosyphilis.

#### Other Nervous System Diseases

One study reported syphilis serological test results among 36,151 patients hospitalized for nervous system disorders; only one neurosyphilis patient was identified among 449 patients with reactive syphilis serological tests (21). Another study recruited 70 HIV-infected patients with nervous system disorders, and only one patient (1.4%) was diagnosed with neurosyphilis (22). These two patients were diagnosed without any evidence on CSF tests.

#### Syphilis Patients With Neurological Symptoms or Who Received Lumbar Puncture

Three retrospective studies reported neurosyphilis among syphilis patients with neurological symptoms. The prevalence of neurosyphilis was 23.7% (100/422), 33.2% (123/370), and 35.5% (156/440) (23–25), respectively. All neurosyphilis patients were diagnosed according to reactive CSF serological tests. Four retrospective studies reported patients with suspected neurosyphilis who underwent lumbar puncture (LP). The prevalence of neurosyphilis in these studies ranged from 13.6 to 61.2% (26–29), respectively. However, only 67.72% (426/630) of the patients were diagnosed with neurosyphilis according to reactive CSF serological tests; the remaining 204 patients had only abnormal routine CSF test results.

#### Serofast Status and RPR/TRUST Titer Fluctuations After Treatment

Six retrospective studies reported 1,171 serofast syphilis patients and 44 patients with RPR/TRUST titers that fluctuated continuously (30–35). A total of 405 (33.3%) patients were diagnosed with neurosyphilis according to reactive results in CSF serological tests and/or abnormal results on CSF routine tests. The prevalence of neurosyphilis in these studies ranged from 9.8 to 56.1%. Unfortunately, 99 neurosyphilis patients were diagnosed without CSF serological evidence, and one study did not provide the results of CSF serological tests.

#### Individuals With HIV and Syphilis Coinfection

A retrospective study recruited 157 patients with HIV and syphilis coinfection, and 8.9% (14/157) of them were diagnosed with neurosyphilis according to reactive CSF-VDRL (seven cases) or CSF-WBC >20 cells/mL (seven cases) (36). Another study recruited 102 syphilis patients coinfected with HIV and with abnormal CSF routine tests, and half of patients were diagnosed with neurosyphilis according to reactive CSF-TPPA with/without reactive CSF-RPR (15 cases) (37).

#### Malignant Syphilis

A retrospective study reported 26 malignant syphilis patients with typical, serious skin lesions and high non-treponemal tests titers (38). Seven (26.9%) patients were diagnosed with neurosyphilis according to the reactive CSF-VDRL test among these patients. The study found that the proportion of malignant syphilis patients who developed concurrent neurosyphilis was higher than common syphilis patients (13.1%).

#### Other Studies

A retrospective study reported an 1.3% prevalence of neurosyphilis in 1,927 inpatients with positive syphilis screening results (39). Another study reported an 82.1% prevalence of neurosyphilis in 1,968 tertiary syphilis and only 27.1% of neurosyphilis patients received standard treatment (5). Recently, a study reported a 78.8% prevalence of neurosyphilis in 528 laboratory-confirmed syphilis in department of neurology or HIV infection patients (40). Another two studies reported 13.7% and 23.6% prevalence of neurosyphilis in 73 and 512 patients with serofast status, or coinfection with HIV, or with neurological symptoms, or serum titer fluctuation after treatment, respectively (41, 42).

### Ocular Syphilis and Otic Syphilis

Four case reports included 6 ocular syphilis patients, and 11 retrospective case series included 244 patients (Supplementary Table S4) which comprised 163 males and 81 females, indicating that ocular syphilis tended to occur more frequently in males than in females (|Z| = 2.56, *P* = 0.01). LP was not performed in two case reports and four retrospective case series; 91.9% (136/148) of the patients in the remaining nine studies received LP, and 56.1% (78/139) (ranged from 9.1 to 91.7%) of them were diagnosed with neurosyphilis according to the reactive CSF serological tests.

A retrospective case series analysis reported 6 syphilis patients with recurrent refractory vertigo and sensorineural deafness. Only one patient with CNS symptoms underwent LP and was diagnosed with neurosyphilis according to the reactive CSF serological tests. (Supplementary Table S4).

### Diagnostic Criteria

The CSF diagnostic criteria differed among 93 retrospective case series studies and 26 studies on neurosyphilis amongst other diseases (Table 6), 42 of which mentioned that neurosyphilis was diagnosed according to US CDC or/and European guidelines, and 25 of which mentioned diagnosed according to Chinese guidelines. It was worthy to note that most studies (84.0%, 100/119) met diagnostic criteria established by the Chinese National Guidelines (14), which included VDRL/RPR/TRUST and/or the FTA-ABS/TPPA/TPHA positivity for CSF samples, as well as abnormal CSF-WBC or CSF-PRO levels. Unfortunately, eight studies (6.7%) did not present the results of routine CSF tests. Two studies diagnosed neurosyphilis with only TPPA/TPHA positivity in CSF. Three studies defined neurosyphilis as reactive CSF-RPR/TRUST or CSF-TPPA/TPHA or abnormal CSF-WBC or CSF-PRO levels. The diagnostic criteria were not mentioned in six studies.

**Table 6:** Diagnostic criteria in 93 retrospective case series and 26 studies on neurosyphilis in patients with other diseases.

**Criteria for the diagnosis of neurosyphilis**	**Number of studies**
VDRL/RPR/TRUST and/or FTA-ABS/TPPA/TPHA positivity and abnormal CSF-WBC or PRO levels	100
VDRL/RPR/TRUST and/or FTA-ABS/TPPA/TPHA positivity and no evidence of CSF-WBC or PRO level abnormalities	8
Only TPPA/TPHA positivity, with no evidence of CSF-WBC or PRO level abnormalities	2
RPR/TRUST positivity, or TPPA/TPHA positivity, or CSF-WBC or PRO level abnormalities	3
Not mentioned	6

*NS, neurosyphilis; VDRL, venereal disease research laboratory; RPR, rapid plasma reagin; TRUST, toluidine red unheated serum test; FTA-ABS, fluorescent treponemal antibody adsorption; TPPA, treponema pallidum particle agglutination; TPHA, treponema pallidum hemagglutination assay; CSF, cerebrospinal fluid; WBC, white blood cell; PR, protein*.

## Discussion

This is the first review of the literature on the clinical epidemiological characteristics of neurosyphilis in China from 2009 to 2021, as well as neurosyphilis in patients with other associated diseases. The inconsistent diagnostic criteria in these studies and the heterogeneity among case sources led to limited conclusions.

Although the incidence of neurosyphilis in China is unknown, the number of reported cases increased over 13 years according to the results of this review (Supplementary Figure S1), indicating an increasing health threat requiring neurosyphilis prevention and control. The regional distribution of cases showed a concentration in the eastern coastal areas, including Guangdong, Fujian, Beijing, Zhejiang, Shanghai, etc. (Supplementary Figure S2). YS Tao et al. found that the incidence of early syphilis in inland provinces has increased over time and has been higher than that in eastern coastal provinces since 2010 (2). Considering that the prevalence of neurosyphilis is similar to that of syphilis, more attention probably be paid to the detection and research of neurosyphilis in eastern coastal areas than inland areas. Therefore, more training in the management of neurosyphilis in inland areas is needed in the future.

Among all the studies, we found that neurosyphilis, as well as ocular syphilis, tended to occur most frequently in middle-aged males, consistent with previous reports (27, 43). We also found that parenchymal syphilis was the main manifestation of neurosyphilis, and general paresis with progressively impaired memory, mental abnormalities and occasional seizures was the most common symptom. This clinical spectrum is consistent with those in previous reports in China but is very different from those associated with cases in Western countries (7). In the penicillin era, early forms, such as meningitis and meningovascular, are more common than late forms in Western countries because treatment can effectively prevent the progression of neurosyphilis (44), as indicated in the most recent reports (45). This result suggests that the early detection and treatment of neurosyphilis in China probably be insufficient. In addition, with the rapid expansion of syphilis screening among all populations, an increasing number of patients infected with syphilis have been diagnosed with neurosyphilis, which has probably progressed to the late stage at the time of diagnosis. It is worth noting that many patients present with atypical, ill-defined neurological complaints (46, 47), causing great challenges in the diagnosis of neurosyphilis for clinicians. Unfortunately, the results showed that the misdiagnosis rate of neurosyphilis was more than 50%, and the most common misdiagnosed diseases were neurodegenerative diseases (including stroke, cognitive impairment, Alzheimer's disease, Parkinson's disease, epilepsy, etc.), mental disorders, encephalitis and ophthalmic diseases. Two studies reporting patients with reactive CSF serological tests and diagnosed with neurosyphilis showed that the prevalence rates of neurosyphilis among patients with CNS-associated infectious diseases and CNS disorders and HIV coinfection were 30.1 and 10.6% (16, 19), respectively. In addition, among patients with syphilis infection, the prevalence of neurosyphilis ranged from 23.2 to 35.5% among patients with neurological symptoms (23–25), 13.6 to 67.7% among patients with suspected neurosyphilis who underwent LP (26–29), and 9.8 to 56.1% among syphilis patients with serofast status (30–35); additionally, the rates were 8.9, 26.9 and 43.0% in individuals coinfected with HIV, malignant syphilis and ocular syphilis (36, 37), respectively. The results suggest that clinicians in neurology, psychiatry and ophthalmology departments should pay much attention to detecting suspected neurosyphilis. Serum syphilis tests can be performed in patients with neurological, psychiatric, or ocular symptoms caused by unknown etiologies, especially patients with CNS-associated infectious diseases or HIV infection, to examine the status of syphilis infection. Moreover, CSF serological testing is recommended for syphilis patients with neurological symptoms, serofast status, coinfection with HIV or the presence of serious skin lesions.

Penicillin G has been demonstrated to be effective for the serological and clinical cure of neurosyphilis since 1940 (48, 49), and it has always been the first-line drug for the treatment of neurosyphilis. Ceftriaxone can be used as an alternative regimen in patients with penicillin allergy, and no difference was found in the efficacy of the two drugs in previous studies (50, 51). The data from case reports studies in this paper also showed that there was no difference in the recovery rate of clinical manifestations of patients treated with penicillin or ceftriaxone. It was worthy to note that approximately 4% of patients did not receive treatment regimens recommended by the national guidelines according to the available data in this review, which will be detrimental to preventing the progression of CNS injury. In addition, neurosyphilis patients need long-term follow-up for clinical management according to the recovery of disease, however, the follow-up results within 6 months were reported in more than half of cases. Hence, medical institutions need to strengthening the management of follow-up and increase the compliance, such as call patients regularly to urge return visits, provide higher quality of health care for patients, provide multidisciplinary therapy, psychological guidance, etc. In addition, we found that 81.7% patients had an improvement of symptoms, and 77.1% patients had a decrease of CSF WBC counts during the follow-up period, however, only 66.7% and half of patients had responses to serological tests of syphilis in serum and CSF, respectively. Therefore, more sensitive and specific biomarkers for assessment of prognosis of neurosyphilis are needed, especially if effective biomarkers can be found in serum in patients unwilling to undergo lumbar puncture during follow-up.

From the results of the case reports and retrospective case series, the treponemal test positivity rate was over 95%, which was significantly higher than the non-treponemal test positivity rate (~75%), and abnormal CSF-WBC counts and CSF protein levels were observed in nearly 70% of NS patients, supporting the importance of CSF serological testing for diagnosis (52). Although the CSF-TPPA testis diagnostically sensitive, considering that TP-IgG can cross the intact blood brain barrier (BBB), reactive treponemal testing of CSF samples is not specific for the diagnosis of neurosyphilis (53). The specificity of the CSF-TPPA test has been debated and ranges from 49 to 84% in some studies (54, 55), and its recommendation as a diagnostic indicator varies according to different guidelines (14, 56, 57). Moreover, the cases in most studies were retrospectively analyzed and included reactive treponemal test in the inclusion criteria, which probably biased positivity rate toward 100% (53). Therefore, CSF nontreponemal tests combined with treponemal tests are needed to reduce the possibility of misdiagnosis.

Neurosyphilis patients frequently have abnormal neuroimaging findings due to CNS inflammation and impairment. Neuroimaging may also show progression in patients who have no neurological symptoms or signs and in patients who receive standardized treatment (58). In addition, neurosyphilis patients may have specific EEG signal characteristics that are different from those of non-neurosyphilis patients (59), and the EEG-Lempel-Ziv complexity (LZC) value may be used as a diagnostic index and the reference index to assess neurosyphilis cure (60). According to our review, abnormal neuroimaging or EEG findings were observed in approximately 80% of patients, and the neuroimaging findings of 97% cases improved or return to normal on follow-up, supporting the recommendation of neuroimaging and EEG examinations for the differential clinical diagnosis and neurosyphilis follow-up (61).

Progression to neurosyphilis is more common in patients coinfected with HIV (62). We found that 8.9% of patients with neurosyphilis were infected with HIV, but most studies have used HIV infection as an exclusion or inclusion criterion, resulting in inconsistent rates.

Although the diagnostic criteria in most of the included studies followed international or national guidelines, unlike previous reports from Africa (15), approximately one-tenth of the studies provided insufficient evidence for a neurosyphilis diagnosis. Hence, standardized diagnostic criteria and protocols are urgently needed to ensure accurate diagnostic results and research conclusions.

This review had several limitations. First, there were some missing clinical information extracted from many studies, especially the data of treatment and follow-up, may result in information bias. Second, the great heterogeneity between studies according to the different diagnostic criteria of neurosyphilis may result in limited conclusions. Third, we did not review the literature published before 2009 due to the lack of clinical diagnosis and treatment guidelines for neurosyphilis in China before 2008, may lead to selection bias.

## Conclusions

In summary, through the review of clinical epidemiology data on neurosyphilis in China, we found that the reported cases of neurosyphilis in the literature presented an increasing trend. More than half of the cases were in the late stage of neurosyphilis, suggesting that early detection of neurosyphilis was inadequate. The most common misdiagnosed diseases and the high prevalence of neurosyphilis amongst patients with CNS-associated infectious disease, CNS disease comorbid with HIV, syphilis with neurological symptoms, serofast status, coinfection with HIV or presesntations comprising serious skin lesions should remind clinicians to pay much attention to the early detection of neurosyphilis among these cases. Meanwhile, standardized treatment and long-term follow-up of neurosyphilis according to the national guidelines should be strengthened to promote the recovery of patients. During follow-up, neuroimaging and EEG examinations should be performed, as they play a positive role in auxiliary diagnosis and the observation of curative effects. Moreover, the combination of CSF non-treponemal tests with treponemal tests should be implemented for diagnosis to reduce misdiagnosis. Inadequate diagnostics remain a great obstacle to progress in understanding this disease. Future well-designed prospective studies are needed to better delineate the incidence and clinical epidemiological of neurosyphilis in China.

## Data Availability Statement

The original contributions presented in the study are included in the article/Supplementary Material, further inquiries can be directed to the corresponding author/s.

## Author Contributions

F-ZD, Q-QW, and R-LZ wrote the draft and revised it. F-ZD, H-NZ, J-JL, and Z-JZ selected literature. F-ZD and XZ extracted data from literature. F-ZD performed the statistical analysis. All authors contributed to the article and approved the submitted version.

## Funding

This work was supported by the Union Innovation Team Project of the Chinese Academy of Medical Sciences (2016-I2M-3021), the National Natural Science Foundation of China (81772209 and 81601804), and the Nanjing Incubation Program for National Clinical Research Center (2019060001).

## Conflict of Interest

The authors declare that the research was conducted in the absence of any commercial or financial relationships that could be construed as a potential conflict of interest.

## Publisher's Note

All claims expressed in this article are solely those of the authors and do not necessarily represent those of their affiliated organizations, or those of the publisher, the editors and the reviewers. Any product that may be evaluated in this article, or claim that may be made by its manufacturer, is not guaranteed or endorsed by the publisher.
