# Supplementary figures and images for: Neurosyphilis in China: A Systematic Review of Cases From 2009–2021
Source: Front Med (Lausanne). 2022 May 13;9:894841. doi: 10.3389/fmed.2022.894841 (PMC9136070; doi:10.3389/fmed.2022.894841)

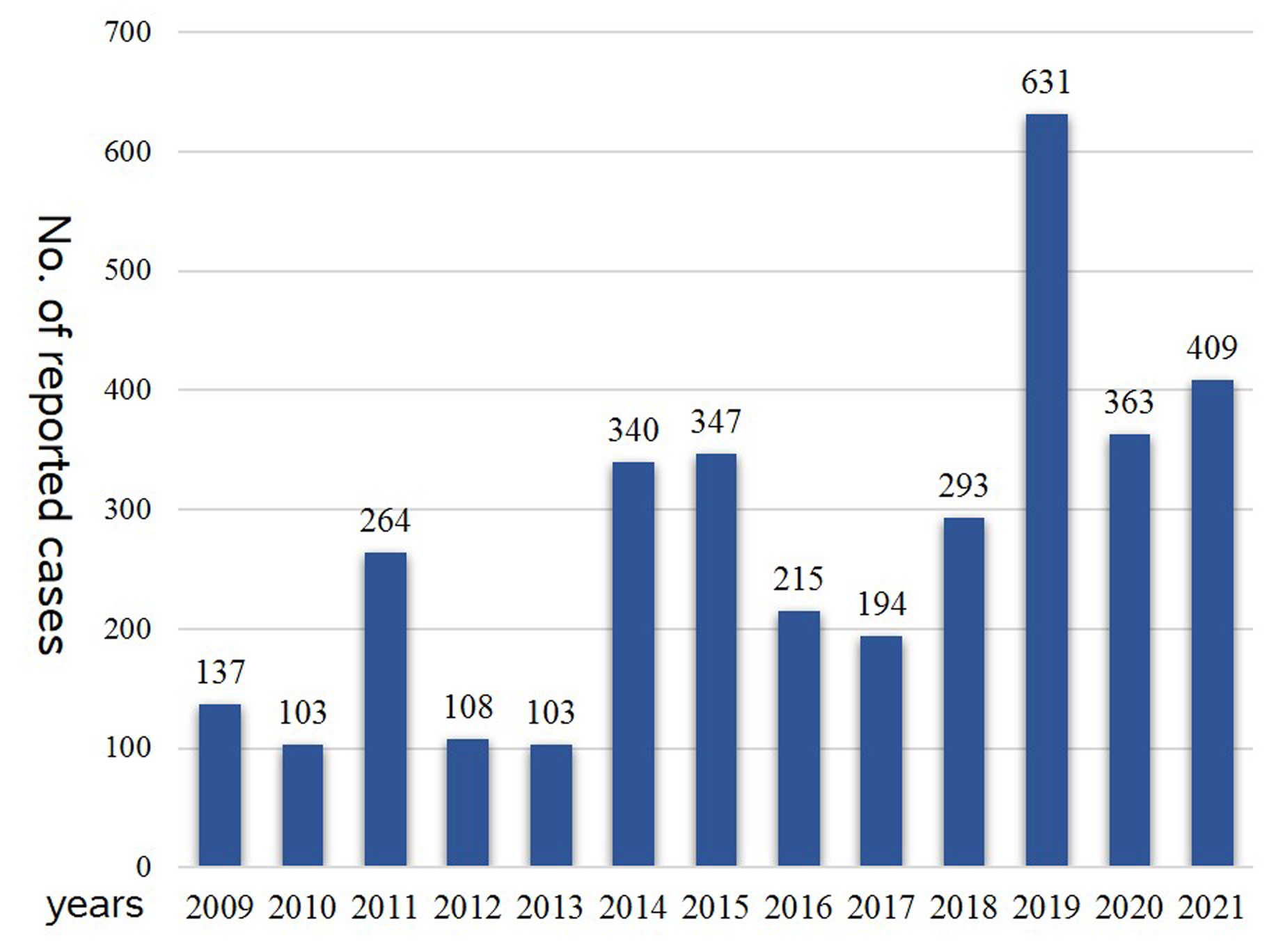

Supplement: Supplementary file 1 [file Data_Sheet_1.ZIP › S Figure1.tif]

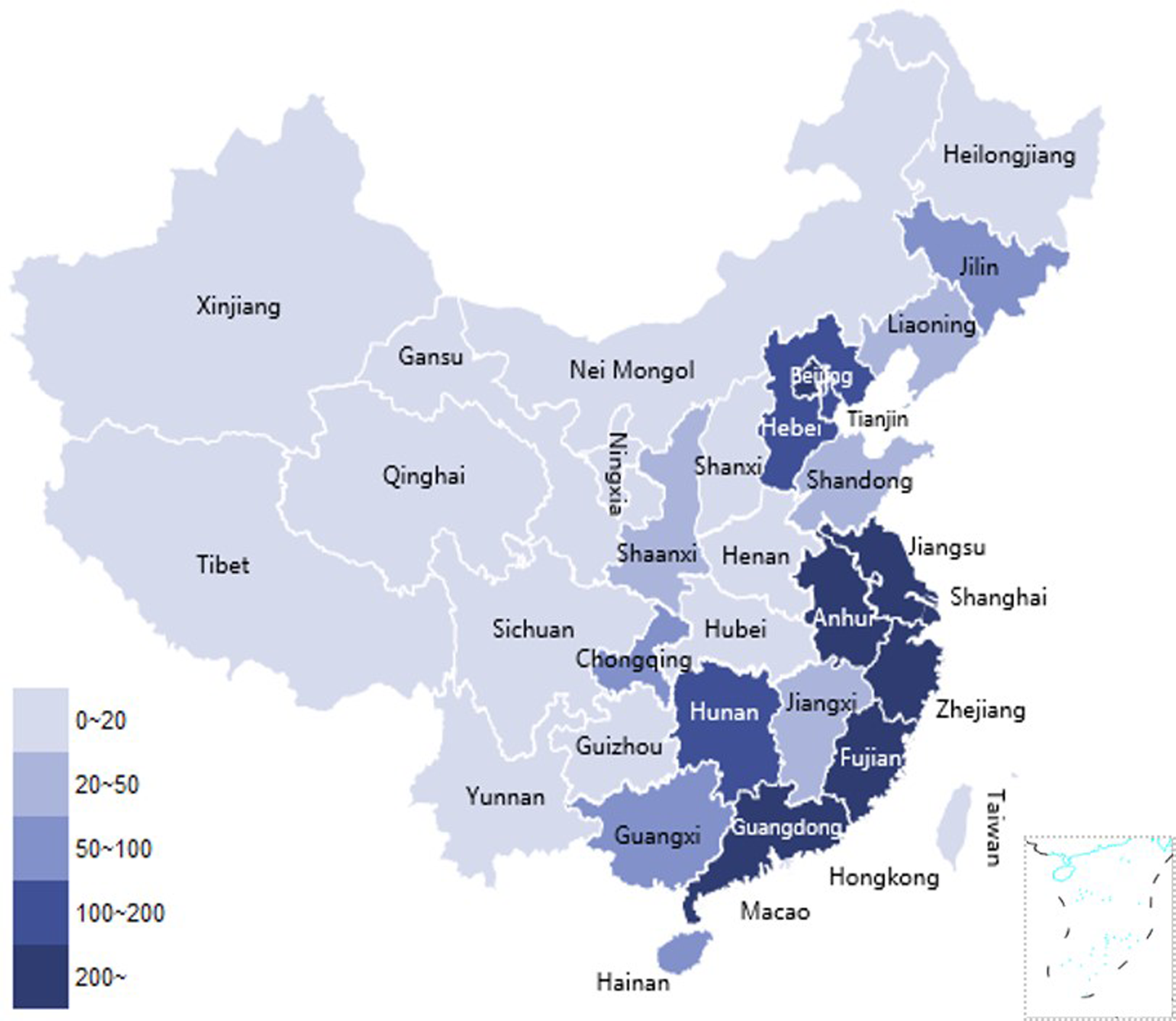

Supplement: Supplementary file 1 [file Data_Sheet_1.ZIP › S Figure2.tif]
